# The Association between Drug-Related Problems and Length of Stay of Type 2 Diabetes Mellitus Patients

**DOI:** 10.21315/mjms2024.31.4.13

**Published:** 2024-08-27

**Authors:** Niken Larasati, Satibi Satibi, Susi Ari Kristina, Lutfan Lazuardi

**Affiliations:** 1 Doctoral Program in Pharmacy, Faculty of Pharmacy, Gadjah Mada University, Yogyakarta, Indonesia; 2 Pharmacy Study Program, Faculty of Health, Jenderal Achmad Yani University, Yogyakarta, Indonesia; 3 Department of Pharmaceutics, Faculty of Pharmacy, Gadjah Mada University, Yogyakarta, Indonesia; 4 Department of Public Health, Faculty of Medicine, Public Health and Nursing, Gadjah Mada University, Yogyakarta, Indonesia

**Keywords:** drug-related problems, length of stay, diabetes mellitus

## Abstract

**Introduction:**

Drug-related problems (DRPs) are treatment-related occurrences that affect therapeutic efficacy. In a previous study, approximately 279 out of 330 (84.5%) patients with type 2 diabetes mellitus (T2DM) had experienced at least one DRP, including non-optimal drug effects (*n* = 240, 52.7%) and indications without medication (*n =* 137, 30.1%). Patients who were hospitalised for 5–10 days had the highest number of DRPs. Therefore, this study investigates the association between DRPs and length of stay (LoS) in patients with T2DM.

**Methods:**

A cross-sectional study was conducted from January 2020 to May 2023 at Rumah Sakit Akademik, Universitas Gadjah Mada, Yogyakarta, Indonesia. Clinical pharmacists reviewed electronic health data to examine DRPs. The Fisher’s exact test evaluated the association between DRPs and LoS.

**Results:**

A total of 60.7% (*n* = 17) of the participants were females, with the majority falling into the age group ≥ 65 years old (*n* = 11, 29.7%). A significant portion experienced LoS > 7 days (*n* = 17, 60.7%). Antidiabetic monotherapy was predominant, and the categories of DRPs included adverse drug reaction (*n =* 15, 40.5%), dosage too high (*n* = 6, 16.2%), wrong drug (*n* = 6, 16.2%), non-adherence (*n* = 4, 10.8%), need for additional therapy (*n =* 4, 10.8%) and dosage too low (*n =* 2, 5.4%). A significant association was observed between non-adherence and LoS (*P =* 0.016). The possibility of experiencing LoS of 1–7 days increased by 3.43 times with improved non-adherence (OR = 3.43; 95% CI: 1.83, 6.39). In this context, non-adherence refers to DRPs associated with the non-compliance of patients with the prescribed treatment plan.

**Conclusion:**

This study concludes that non-adherence was significantly associated with hospital LoS.

## Introduction

Drug-related problems (DRPs) are treatment-related occurrences in patients that affect therapeutic efficacy (1). Pharmacists play a crucial role in managing therapy by applying pharmaceutical care concepts, including assessment, planning, implementation of pharmaceutical service plans and therapy monitoring (2). Clinical pharmacy activities enhance patient safety and address treatment-related problems (3). Previous studies have shown a significant incidence of DRPs in chronic diseases (*n* = 167), including unnecessary therapeutic events (*n* = 58, 34.7%), untreated indicative events (*n* = 114, 68.3%), ineffective therapy (*n* = 125, 74.9%), inappropriate dose (*n* = 84, 50.3%) and adverse drug reactions (ADR) (*n* = 40, 10.2%) (4). Additionally, 41 out of 46 patients (89.1%) are at risk of having treatment-related problems (5). Patients with type 2 diabetes mellitus (T2DM) experienced 126 DRPs (80.8%), with the most common issues being the need for additional therapy (*n* = 60, 40.3%), lack of compliance (*n* = 51, 34.2%) and unnecessary therapy (*n* = 12, 8%) (6). Sheleme et al. (7) reported that 279 out of 330 (84.5%) patients with T2DM had at least one DRP, including non-optimal drug effects (*n* = 240, 52.7%) and indications without medications (*n* = 137, 30.1%). These problems significantly impact the anticipated treatment outcomes for T2DM.

According to the International Diabetes Federation (IDF), the global prevalence of DM reached 463 million in 2019, with Indonesia ranking 7th with 10.6 million patients. The worldwide prevalence is expected to increase to 578 and 700 million in 2030 and 2045, respectively, marking a 51% rise (8). In Indonesia, the death rate due to diabetes ranks 2nd after Sri Lanka (9). As per the Basic Health Research/Riset Kesehatan Dasar (RISKESDAS) (10), Yogyakarta Special Region Province ranked 3rd for diagnosed DM cases. Treatment modalities include oral antidiabetic drugs, insulin injections or a combination of both, depending on the doctor’s diagnosis (10).

Movva et al. (11) observed that patients with length of stay (LoS) ranging 5 days–10 days had the highest incidence of DRPs. These problems contribute to increased treatment costs and impact the LoS due to therapeutic inefficacy (12). Previous reports investigated the influence of DRPs on LoS in patients with hypertension in pregnancy, revealing a significant increase (13). The present study aimed to determine the association between DRPs and LoS in patients with T2DM.

## Methods

### Study Design, Location and Duration

A cross-sectional study was conducted at Rumah Sakit Akademik, Universitas Gadjah Mada, Yogyakarta, Indonesia, from January 2020 to May 2023 (since clinical pharmacists do integrated documentation in electronic health record or EHR).

### Study Sample and Patient Selection

The study comprised all patients diagnosed with T2DM. The inclusion criteria comprised individuals ii) aged 18 years old and above, ii) patients with T2DM as a primary diagnosis, iii) patients with blood glucose tests, and iv) patients who were actively prescribed antidiabetic medication. Those with incomplete records or missing data were excluded. Determining T2DM as the primary diagnosis relied on International Classification of Disease, 10th Revision (ICD-10) codes assigned by the medical records department.

### Data Collection and Identification of DRPs

Clinical and demographic variables, such as gender, age in years, LoS and type of medications used, were collected along with comorbidity information. This study examined DRPs using electronic health data reviewed by clinical pharmacists. Upon hospitalisation, the clinic’s pharmacist determined the presence or absence of DRPs. During hospitalisation, the presence of DRPs was assessed and the identification results were recorded on the integrated patient progress record sheet within the EHR.

Data related to DRPs were collected by the clinical pharmacists, including current issues, potential occurrences and recommendations offered. The data was extracted and classified into the DRPs category using the classification system of Cipolle, which comprises the following: need for additional therapy, unnecessary therapy, wrong drug, dosage too low, ADR, dose too high and non-adherence (2). The term non-adherence refers to DRPs linked to non-compliance of patients with the prescribed treatment plan.

### Statistical Analysis

Descriptive statistics were used for data analysis, presenting patients’ characteristics and DRPs as numbers and percentages. The Fisher’s exact test evaluated the association between DRPs and LoS. The odds ratio (OR) quantified the strength of the association, with an OR < 1.00 suggesting a reduced probability of LoS of 1–7 days due to DRPs. OR = 1 showed no association, while an OR > 1 suggested a higher probability of LoS of 1–7 days. The significance of this association was determined by a *P* < 0.05, with a 95% confidence interval (CI). Data analysis was performed using the SPSS version 12.0.

## Results

### Demographic Characteristics

**Figure 1:**
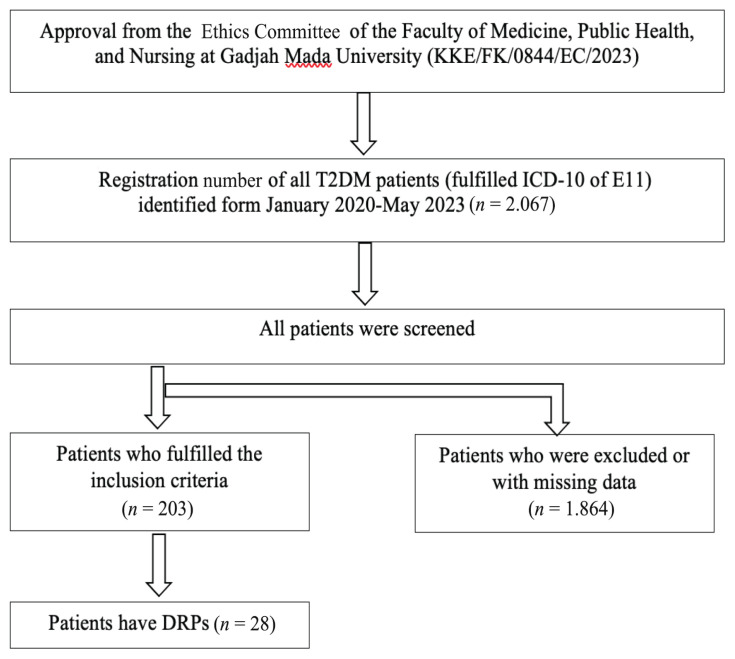
Flowchart of the patient selection

A total of 203 patients with T2DM were initially included but only 28 patients were identified to have at least one DRP and were subsequently analysed (Figure 1). Table 1 shows that out of these 28 patients, 39.3% were males (*n* = 11) and 60.7% were females (*n* = 17). The majority of patients were in the age group ≥ 65 years old (*n* = 11, 29.7%), followed by the age groups of 55 years old–64 years old (*n* = 9, 24.3%), 45 years old–54 years old (*n* = 5, 13.5%) and 18 years old–44 years old (*n* = 3, 8.1%). In terms of their LoS, the majority of patients were hospitalised for more than 7 days (*n* = 17, 60.7%). Additionally, most patients received antidiabetic monotherapy (*n* = 20, 71.4%). The prevalent additional health problems included three comorbidities (*n* = 9, 32.1%). Table 3 represents the details discussed above.

### Medication Used in T2DM Patients and Comorbidities

In this study, most prescribed antidiabetic medications were monotherapy, as shown in Table 2. Rapid-acting insulin was the most commonly used class of drugs (*n* = 11, 29.7%). The most frequently prescribed antidiabetic combinations were rapid-acting insulin + long-acting insulin (*n* = 3, 8.1%) and biguanide + rapid-acting insulin + long-acting insulin (*n* = 1, 2.7%). Additionally, thiazolidinedione + dipeptidyl peptidase-4 (DPP-4) inhibitors + rapid-acting insulin (*n* = 1, 2.7%) was observed. Table 3 provides details of various comorbidities, with cardiovascular diseases being the most common, including congestive heart failure (CHF) (*n* = 4, 3.5%), hyperlipidemia (*n* = 2, 1.8%) and hypertension (*n* = 11, 9.7%).

### Drug-Related Problems

The study identified several categories of DRPs, as represented in Table 4. These included ADR (*n* = 15, 40.5%), dosage too high (*n* = 6, 16.2%), wrong drug (*n* = 6, 16.2%), non-adherence (*n* = 4, 10.8%), need for additional therapy (*n* = 4, 10.8%) and dosage too low (*n* = 2, 5.4%). Non-adherence issues included irregular medication intake, patients’ disinterest in medication and discomfort with specially prepared medication such as insulin. The most prevalent category of DRPs was ADR, including long-term drug use (*n* = 1, 2.7%), potential drug interactions (*n* = 11, 29.7%), alterations in laboratory results due to the use of other drugs (*n* = 1, 2.7%) and the use of drugs with a high-risk profile (*n* = 2, 5.4%). Clinical pharmacists have identified potential drug interactions that require monitoring or follow-up to prevent complications.

### Factors that were Significantly Associated inT2DM with Drug-Related Problems

Table 5 shows a significant association between non-adherence and LoS (*P* = 0.016). The possibility of experiencing LoS of 1–7 days increased by 3.43 times with improved non-adherence (OR = 3.43; 95% CI: 1.83, 6.39).

## Discussion

The occurrence of DRPs has been associated with prolonged hospital stay, increased financial burden, and nearly a two-fold higher risk of mortality (14). Due to the considerable health and financial costs, hospitalisation due to DRPs is a significant concern to both patients and healthcare providers (15). Maximising medication efficiency and preventing these problems is crucial for improving healthcare, reducing expenses and potentially saving lives (16). DRPs are assumed to be expensive, serious and complicated issues for the healthcare system, often associated with polypharmacy, multimorbidity and advancing age in diabetic patients. Risk factors for these problems in diabetic patients also include renal impairment, inadequate cholesterol management, cardiovascular disease and LoS (17).

DRP development was significantly associated with females. The World Health Organization (WHO) (18) offered a compelling explanation, suggesting that females are more prone to being overweight, obese and physically inactive. Additionally, the higher occurrence of poor glycaemic control in females may be linked to biological and psychosocial factors (19, 20). Despite several reports suggesting that older age (> 60 years old) poses a risk for DRPs (21), this study did not find a statistically significant association. The increased occurrence of these problems in geriatric patients might be explained by the correlation between lower creatinine clearance (CrCL), high polypharmacy and a higher number of DRPs (22). Aging is generally associated with an increased risk of ADR and other related issues due to slowed metabolism and excretory processes. Numerous studies have stated that elderly patients taking multiple medications are more susceptible to DRPs (23).

This study shares similarities with Bathari et al. (24), who showed rapid-acting insulin was the most commonly prescribed antidiabetic. Insulin is the ideal option for precise blood glucose regulation, allowing for prompt adjustments based on glucose readings. To prevent hypoglycaemia, insulin therapy generally begins with a small dose. This may include oral medication in addition to insulin or an insulin combination therapy (25).

Consistent with other studies, drug classes frequently linked to DRPs included gastrointestinal, endocrine and cardiovascular medications (26). According to investigations conducted in the UK, Saudi Arabia (27) and Wolaita Sodo, Ethiopia (28), a higher number of comorbidities was linked to an elevated risk of developing at least one DRP. This relationship may arise from individuals with more comorbidities being more prone to taking multiple medications, which may lead to non-adherence and an increased susceptibility to ADR.

A study by Sharma et al (29) revealed a significant association between comorbidities and DRPs. The use of multiple drugs can result in drug-drug interactions and a complex medication schedule. The frequent administration of medication and an increased number of pills may contribute to the occurrence of DRPs.

In this study, ADRs were the most prevalent DRPs. The possibility of encountering these problems increased due to multiple therapies, particularly when six or more different types of medications were involved. This showed the importance of preventing and managing drug interactions (30). Patients with comorbidities, particularly those receiving seven or more medications (polypharmacy), were found to be at a higher risk of ADRs and drug interactions (31).

Studies conducted in Northern Sweden stated that patients admitted to hospitals without clinical pharmacist services frequently experienced inappropriate drug use and drug-drug interactions (32). Drug interactions and dosage issues were identified as frequent DRPs (33). Consistent with a German study (34), where inappropriate medication use and non-adherence problems were also prevalent. Additionally, the LoS was associated with potential drug interactions (35).

DRPs have a significant impact on the quality of life of hospitalised patients, resulting in prolonged hospital stays, higher healthcare expenses and mortality (36). According to a retrospective analysis, DRPs related to non-adherence and adverse effects had the highest potential for clinical significance and risk of harm (37). Additionally, a systematic review and meta-analysis identified a significant relationship between poor medication adherence and the incidence of these problems (38).

Patients experiencing ADR and drug interactions, as shown by Kurniawati et al. (39), tend to have longer LoS. An extended LoS not only requires more hospital resources but also increases the costs of treatments (40). Additionally, another study showed that longer hospitalisations were more prone to have at least one type of DRP, potentially due to an increased risk of nosocomial infections requiring extensive therapeutic interventions (41). Non-adherence issues further compound the challenge, hindering the achievement of therapeutic goals and potentially leading to prolonged LoS.

Increased LoS manifests the challenges faced by inpatients due to medical intervention (42). Hospital pharmacists play a crucial role in mitigating this negative impact, although this task poses a significant challenge, given the demanding workload in hospitals (43). This implied that emphasis should be placed on prioritising patients at the highest risk and in need of guidance (44). Several independent factors contribute to medication-related harm during hospitalisation, including advanced age, comorbidities, impaired kidney function and the use of high-risk medications (45). In addition to taking more prescription drugs, patients with multiple chronic conditions are more to experience medication-related issues. In older adults, physiological changes associated with aging affect drug pharmacokinetics and pharmacodynamics, leading to a higher risk of adverse medication events. Additionally, comorbidities and advanced age can contribute to polypharmacy and an increased risk of non-adherence (46). Table 5 shows patients without compliance problems had a longer LoS (> 7 days). Pharmacists agreed that the risk of experiencing an adverse medication event increased with duration of hospitalisation. Consequently, LoS is one of the priority criteria for clinical pharmacist therapy monitoring. Pharmacist interventions improved medication adherence in most studies. The interventions by pharmacists include the evaluation of medications, delivering educational sessions and counseling, and offering therapeutic suggestions to prescribing healthcare professionals (47–49). Additionally, patients with longer LoS were stated to have a lower possibility of medication changes. To achieve optimal outcomes and enhance medication safety, it is essential to promptly identify high-risk cases and proactively prevent or minimise drug-related issues (44).

An insightful study conducted in Pakistan emphasised the importance of keeping clinical knowledge of pharmacists up-to-date to improve their ability to make interventions that effectively lower the incidence of DRPs (50). In the future, modern technology can be leveraged to both prevent and identify DRPs, contributing to enhanced safety of patients and the achievement of therapeutic goals.

### Strengths and Limitations of the Study

The strength of this study lies in the identification of DRPs using the classification system of Cipolle, a recognised method employed by clinical pharmacists. However, the study is limited by its small sample size. Additionally, the performance of pharmacist intervention was not evaluated. Future research requires a more extensive confirmatory and multicentre study to overcome these limitations.

## Conclusion

In conclusion, ADRs were the majority of DRPs identified in this study. Additionally, a significant association between non-adherence and extended hospital LoS was observed. Pharmacists play an essential role in influencing the outcomes of T2DM patient management by proactively preventing DRPs through pharmaceutical care.

## Figures and Tables

**Table 1:** Demographic characteristics of the patients

Variable	Frequency *n* (%)
Gender	
Male	11 (39.3)
Female	17 (60.7)
Age	
18−44	3 (8.1)
45−54	5 (13.5)
55−64	9 (24.3)
≥ 65	11 (29.7)
LoS	
1−7 days	11 (39.3)
> 7 days	17 (60.7)
Antidiabetic drug	
Monotherapy	20 (71.4)
2 combination antidiabetic	6 (21.4)
3 combination antidiabetic	2 (7.1)
Comorbid*	
1 comorbid	5 (17.9)
2 comorbid	5 (17.9)
3 comorbid	9 (32.1)
4 comorbid	4 (14.3)
5 comorbid	4 (14.3)
6 comorbid	1 (3.6)

Note:

* comorbidity

**Table 2:** Distribution of the type of prescribed antidiabetic

Antidiabetic	Frequency *n* (%)
Monotherapy	
Sulfonylurea	3 (8.1)
Thiazolidinedione	1 (2.7)
Rapid-acting insulin	11 (29.7)
Long-acting insulin	2 (5.4)
Fast-acting insulin	3 (8.1)
Two combination antidiabetic	
Two combination rapid-acting insulin	1 (2.7)
Rapid-acting insulin + long-acting insulin	3 (8.1)
Alfa glucosidase inhibitor + inhibitor dipeptidyl peptidase-4 (DPP-4)	1 (2.7)
Biguanid + inhibitor dipeptidyl peptidase-4 (DPP-4)	1 (2.7)
Three combination antidiabetic	
Biguanid + rapid-acting insulin + long-acting insulin	1 (2.7)
Thiazolidinedione + inhibitor dipeptidyl peptidase-4 (DPP-4) +rapid-acting insulin	1 (2.7)

**Table 3:** Distribution of the type of comorbidities

Type of comorbidity	Frequency *n* (%)
Gastrointestinal disease	
Dyspepsia	2 (1.8)
Nausea vomiting	1 (0.9)
Cholecystitis	1 (0.9)
Electrolyte balance disturbance	
Hyperkalemia	1 (0.9)
Hypo-osmolality and hyponatremia	7 (6.1)
Hypokalemia	2 (1.8)
Respiratory disorders	
CPOD	1 (0.9)
Pneumonia	1 (0.9)
Cardiovascular disease	
CHF	4 (3.5)
Hyperlipidemia	2 (1.8)
Hypertension	11 (9.7)
Anaemia	8 (7.0)
Stroke	3 (2.6)
Diabetic ulcer	2 (1.8)
Psychiatric disorders	
Anxiety disorder	1 (0.9)
Schizoaffective disorder	1 (0.9)
Kidney disease	
CKD	11 (9.7)
Nephrotic syndrome	1 (0.9)
Extracorporeal dialysis	1 (0.9)
Others	23 (20.2)

**Table 4:** Distribution of the classification of DRPs

DRPs category	Proportion *n* (%)
Adverse drug reaction	
Long-term drug used	1 (2.7)
Potential drug interaction	11 (29.7)
Changes in laboratory results due to the use of other drugs	1 (2.7)
Use of drugs with a high-risk profile	2 (5.4)
Dosage too high	
Patients need dosage adjustments for kidney disease	
Ketoconazole	1 (2.7)
Gentamycin	2 (5.4)
Levofloxacin	1 (2.7)
Ceftriaxone	1 (2.7)
Fenofibrate	1 (2.7)
Wrong drug	
Patients with risk factors for contraindications	5 (13.5)
Patients receive a drug that is not the most effective for their indication	1 (2.7)
Non-adherence	
Discomfort associated with insulin use	2 (5.4)
Patient feels bored	1 (2.7)
Patient forgets not to take	1 (2.7)
Need additional therapy	
Due to lab results	2 (5.4)
Untreated condition	2 (5.4)
Dosage too low	
The patient received a dose that was too low to respond	
Atorvastatin	1 (2.7)
Meropenem	1 (2.7)

**Table 5:** The associate between DRPs and LoS in T2DM patients

DRPs	LoS		Proportion *n* (%)	*P*-value	OR (CI 95%)

	1–7 days	> 7 days			
Dose too high					
Yes	2	4	6 (21.4)	1.000	0,72 (0.11, 4.82)
No	9	13	22 (78.6)		
Adverse drug reaction					
Yes	3	9	12 (42.9)	0.253	0.33 (0.06, 1.70)
No	8	8	16 (57.1)		
Dose too low					
Yes	0	2	2 (7.1)	0.505	1.73 (1.25, 2.40)
No	11	15	26 (92.9)		
Wrong drug					
Yes	3	3	6 (21.4)	0.653	1.75 (0.28, 10.81)
No	8	14	22 (78.6)		
Non-adherence					
Yes	4	0	4 (14.3)	0.016	3.43 (1.83, 6.39)
No	7	17	24 (85.7)		
Need additional drug					
Yes	1	3	4 (14.3)	1.000	0.47 (0.04, 5.17)
No	10	14	24 (85.7)		
